# Editorial: New advances in the biology and pathogenesis of free-living amoebae

**DOI:** 10.3389/fmicb.2024.1401217

**Published:** 2024-04-29

**Authors:** Isabel Marcelino, Ascel Samba-Louaka, Christopher A. Rice

**Affiliations:** ^1^Institut Pasteur de la Guadeloupe, Les Abymes, Guadeloupe, France; ^2^Laboratoire Ecologie et Biologie des Interactions, Université de Poitiers, Unité Mixte de Recherche (UMR) Centre national de la recherche scientifique (CNRS) 7267, Poitiers, France; ^3^Department of Comparative Pathobiology, College of Veterinary Medicine, Purdue University, West Lafayette, IN, United States; ^4^Purdue Institute for Drug Discovery (PIDD), Purdue University, West Lafayette, IN, United States; ^5^Purdue Institute of Inflammation, Immunology and Infectious Disease (PI4D), Purdue University, West Lafayette, IN, United States

**Keywords:** free-living amoebae, pathogenesis, FLAM 2023, *Naegleria*, *Acanthamoeba*, *Balamuthia*, reviews, novel methods

## Introduction

Free-living amoebae (FLA) are fascinating unicellular eukaryotic microorganisms found worldwide in aquatic and soil habitats. They have an important role in the ecosystems, actively feeding mainly on bacteria and other microorganisms. FLA life cycle is mainly composed of two stages: the trophozoite (the metabolically and replicative form of the amoeba) and the persistent cyst (which is highly resistant to various adverse conditions such as water disinfection processes and therapeutic treatments). While being mainly non-pathogenic, some FLA (namely *Acanthamoeba* and *Naegleria fowleri*) are currently considered emerging opportunistic pathogens (Bartrand et al., 2014; Sente et al., 2016). FLA are also well-known reservoirs of amoeba-resistant bacteria (ARB), possibly contributing to the spread of pathogenic ARB (such as *Legionella*), which constitutes a potential threat to water quality and human health (Samba-Louaka et al., 2019; Chaúque et al., 2022). It is therefore crucial to increase awareness of these neglected waterborne pathogens and related diseases. This Research Topic presents recent research on several FLA topics and includes studies presented by participants who attended the 19th International Free-Living Amoebae Meeting (FLAM) held in Poitiers, France, in June 2023.

## Summarizing the papers accepted

In this Research Topic, *Naegleria fowleri* extracellular vesicles (EVs) have garnered great interest in our community, with two papers by independent lab groups being published. Russell et al. and Retana Moreira et al. have both investigated EV characterization and their effect on various clinical isolates of *Naegleria fowleri*, as well as on host cells such as B103 neuroblastoma or primary cultures of mouse cell microglia, using SEM methods. Staying on the theme of *N. fowleri*, Nadeem et al. highlight the emerging threat and “outbreak” of *N. fowleri* in Pakistan. This review highlights the need for improved awareness, public health measures, and water surveillance. Dereeper et al. contributed six new high-quality *Naegleria* genomes to establish the *Naegleria* genus pangenome with a near-to-complete repertoire of core and accessory genes, highlighting new architecture and functional features in *Naegleria*.

Excitingly, there was a description of one new putative host species of mycobacteria, *Rosculus vilicus*, within the environment that was published by Jessu et al.. This may pose as a potential host and transmission route for Johne's disease and should be kept a close eye on in the future.

A review from Wang et al. describes the characteristics of *Acanthamoeba* infection, including biological characteristics, classification, disease, and pathogenic mechanism, in order to provide a scientific basis for the diagnosis, treatment, and prevention of *Acanthamoeba* infection.

Loufouma Mbouaka et al. used Realtime-Glo as a novel two-dimensional (2D) cytotoxicity viability assay, being able to monitor the cell health and the pathobiology of *Acanthamoeba* on feeder cells. This assay could be adapted for *Balamuthia* pathogenicity models as well, but because *Naegleria* can also metabolize the propriety reagent, it may be difficult to differentiate between the pathogen or host response in that model.

One study assessed the phylogenetic diversity of the mitochondrial genome ribosomal protein S3 (rps3) over 10 strains of *B. mandrillaris*. Law et al. proposed that due to the copy-number variations (CNVs) and highly variable sequences of the protein tandem repeats of rps3, this could be a perfect target for a clinical genotyping assay for *B. mandrillaris*.

Whangviboonkij et al. presented a three-dimensional (3D) human neurospheroid model to assess the cytotoxicity and pathobiology response of *B. mandrillaris*. Using 3D models that mimic the human central nervous system (CNS) can provide a more physiologically relevant environment than traditional 2D cell culture for studying the pathogenicity of amoeba. These models are particularly useful for reducing the need for experiments using animal models.

Sticking to the theme of 3D models, Campolo et al. describe the aggregation and encystment of *Acanthamoeba*'s response on various contact lens materials under the same conditions. They found that some lenses will promote this aggregation phenotype, which will induce rapid encystment of some *Acanthamoeba* cells within a few hours to fully mature cysts, which they believe helps *Acanthamoeba* to withstand the disinfection process of contact lens care solutions.

Finally, to bring all the amoebae together, Ferrins et al. describe novel chemical pharmacophores that have various inhibitory activities against *Acanthamoeba* sp., *Naegleria fowleri*, or *Balamuthia mandrillaris*, which have been shown to cross the blood–brain barrier. This exciting physiological property is much needed for the development of any future anti-amoebic therapeutics for CNS disease. We cannot just stop at showing the amoeba inhibitory activity and stating that *in vivo* pharmacokinetic/pharmacodynamic or *in vivo* efficacy models are necessary to “validate the compounds future potential.”

## Conclusion

In conclusion, this Research Topic brings together diverse examples of the ongoing research on FLA regarding (i) FLA biology and pathogenesis (such as *Naegleria* pangenome or *Balamuthia mandrillaris'* mitochondrial heterogeneity), the role of extracellular vesicles in *N. fowleri*–host interaction, (ii) FLA as hosts of zoonotic bacteria, (iii) drug development against FLA (namely brain permeable therapeutics against *Acanthamoeba, Naegleria*, or *Balamuthia*), various newly described 2D and 3D pathobiological models for brain organoid, cytotoxicity, or *Acanthamoeba* aggregation models, and (iv) FLA epidemiology. While this surely represents a glimpse of the ongoing research worldwide, we believe that increased research in disease, epidemiology, diagnostics, and treatment of pathogenic FLA species and the characterization of other emerging FLA should be encouraged, namely within the One Health concept.

Furthermore, we expect the emergence of the use of artificial intelligence (AI) in amoebae research. Indeed, the development and application of machine learning (ML) in the field of infectious diseases have gained massive attention in recent years, including other protozoans such as *Plasmodium* and *Trypanosoma* (Hu et al., 2022). We caught a glimpse of machine learning with Dr. Rice's (unpublished data) and Dr. Debnath's (Shing et al., 2022) novel *Acanthamoeba* cysticidal methodologies at the recent FLAM 2023 conference. Although the European Parliament and the US administration are trying to impose obligations for general-purpose AI to mitigate possible risks to health, fundamental rights, and democracy, we are assisting in the boost of AI in research and innovation. How AI will impact research in free-living amoebae is worth investigating. Beyond the identification of trophozoites or cysts, will AI be able to predict genera of amoebae present within the water or soil during sampling campaigns? Regarding the virulence factors identified in some pathogenic amoebae, will AI suggest human outcomes or some area to investigate, interrogate, and suggest optimal patient-specific treatment options? How deep will this rapidly advancing generative AI turn our field upside down? The next FLAM to be held in Mexico City, Mexico, in 2025, could be a place for an exchange around the impact of AI on the study of amoebae.

## Author contributions

IM: Writing—original draft, Writing—review & editing. AS-L: Writing—original draft, Writing—review & editing. CR: Writing—original draft, Writing—review & editing.

## References

[B1] Bartrand T. A. Causey J. J. Clancy J. L. (2014). Naegleria fowleri: An emerging drinking water pathogen. J. Am. Water Works Assoc. 106, E418–E432. 10.5942/jawwa.20l4.106.0140

[B2] Chaúque B. J. M. dos Santos D. L. Anvari D. Rott M. B. (2022). Prevalence of free-living amoebae in swimming pools and recreational waters, a systematic review and meta-analysis. Parasitol. Res. 121, 3033–3050. 10.1007/s00436-022-07631-3 36040629 PMC9424809

[B3] Hu R. S. Hesham A. E. L. Zou Q. (2022). Machine learning and its applications for protozoal pathogens and protozoal infectious diseases. Front. Cell. Infect. Microbiol. 12:882995. 10.3389/fcimb.2022.882995 35573796 PMC9097758

[B4] Samba-Louaka A. Delafont V. Rodier M. H. Cateau E. Héchard Y. (2019). Free-living amoebae and squatters in the wild: ecological and molecular features. FEMS Microbiol. Rev. 43, 415–434. 10.1093/femsre/fuz011 31049565

[B5] Sente C. Erume J. Naigaga I. Magambo P. K. Ochwo S. Mulindwa J.. (2016). Occurrence and genetic characterisation of Acanthamoeba spp. from environmental and domestic water sources in Queen Elizabeth Protected Area, Uganda. Parasit. Vectors 9:127. 10.1186/s13071-016-1411-y 26935431 PMC4776447

[B6] Shing B. Balen M. Fenical W. Debnath A. (2022). Development of a machine learning-based cysticidal assay and identification of an amebicidal and cysticidal marine microbial metabolite against acanthamoeba. Microbiol. Spectr. 10:e0007722. 10.1128/spectrum.00077-22 35467370 PMC9241814

